# Exploring the complexity of safe insulin management during transfer of care using qualitative methods

**DOI:** 10.1111/dme.70054

**Published:** 2025-05-05

**Authors:** Catherine Leon, Clare Crowley, Helen Hogan, Yogini H. Jani

**Affiliations:** ^1^ Department of Health Services Research and Policy London School of Hygiene & Tropical Medicine London UK; ^2^ Reading School of Pharmacy University of Reading Reading UK; ^3^ Department of Practice and Policy University College London School of Pharmacy London UK; ^4^ Centre for Medicines Optimisation Research and Education University College London Hospitals NHS Foundation Trust London UK

**Keywords:** complications, diabetes, drug safety, insulin

## Abstract

**Aims:**

Managing insulin during care transfers requires improvement. Understanding factors that impact insulin management during this process improves the likely effectiveness of interventions. This study aimed to map the processes involved in managing insulin during transfers of care and the factors that affect them to identify potential areas for safety improvement interventions.

**Methods:**

A qualitative, case study approach was used to undertake documentary analysis, interviews, focus groups and observation. Participants included people with diabetes who use insulin, caregivers and primary and secondary care healthcare professionals. A framework approach guided analysis and subtheme categorisation under the domains of people, tools, tasks or environments.

**Results:**

Insulin management during transfers of care was mapped across hospital admission and discharge along with factors that impact this process. Six stages of the care transfer process were identified. Workforce pressures and demand impacted safe insulin management. Four themes were identified: (1) People with diabetes hold vital information not otherwise available, (2) their ability to manage their diabetes care in hospital was limited, (3) healthcare staff lacked confidence managing insulin and (4) people anticipated and acted to prevent known issues.

**Conclusions:**

A detailed picture of factors impacting insulin management during the transfer of care was developed. Incorporating the expertise of people who use insulin and removing barriers to insulin self management across the care pathway, ensuring staff have adequate knowledge, skills and confidence in the management of insulin and promoting proactive interventions to support safe outcomes represent key interventions to improve safety for people who use insulin.

**What's new**

- *What is already known?* Transfers of care are known to be challenging for safe insulin management for people with diabetes.
- *What has this study found?* Interactions between complex factors impact insulin management during transfers of care. People, including those with diabetes and healthcare professionals proactively maintain safety by anticipating and preventing known issues.
- *What are the implications of this study?* Key interventions to improve safety include incorporating the expertise of people who use insulin during care transfers, ensure staff have insulin management competency and proactive management support.

## 1 INTRODUCTION

When people with diabetes who use insulin (PWDI) move between different parts of the healthcare system, there are many challenges to managing insulin safely. Insulin is a critical and high‐risk medication. It is critical because for many PWDI if not managed correctly, it can cause significant harm, for example, hyperosmolar hyperglycaemic state. It is high‐risk because where an incorrect dose is used unintentionally, the resulting harm can be life threatening.^1^, ^2^ Safety challenges with insulin are well documented. There are a multitude of insulin types and brands that look and sound like each other, which can lead to incorrect insulin prescriptions and administration.^3^ Insulin delays and omissions, more likely on admission and discharge from hospital, can lead to harms such as hyperglycaemia.^4^, ^5^, ^6^ If an incorrect device is used to administer insulin, there is a risk of severe harm due to an incorrect dose being given.^6^, ^7^ Challenges arise due to issues with communication, inadequate involvement of PWDI or their caregivers, and failure to provide support following discharge.^8^ Ensuring insulin is given on time without delays or omissions is also challenging in these transitional periods.^5^, ^9^

Transfer of care (ToC) occurs when a PWDI moves between different care settings and responsibility for the practical and medical management of insulin is transferred. The World Health Organization global safety campaign aimed to reduce harm from high‐risk medications such as insulin during ToC by 2024 through improvement programmes seeking to empower patients, improve access to information and accuracy of medication prescribing following ToC.^9^ There is a need for further research into mechanisms to improve the safe management of insulin during ToC.^10^

Previous safety philosophies have focused on identifying the root cause of harms and introducing policies and fixes to prevent these recurring.^11^ Newer conceptualisations of safety recognise that healthcare is provided within a complex, interacting tangle of factors, known as the work system. These work system factors include people interacting with the tasks they must perform, the equipment being used and the different settings, organisations and legislation under which healthcare is provided.^12^, ^13^, ^14^, ^15^ It is now understood that safety is not just the absence of harm. Instead, safety is thought to be created and maintained by people making necessary adaptations to changing situations created by varying combinations of work system factors.^16^ PWDI and healthcare professionals adjust their activities due to a mismatch between the demands placed on them and the resources available to meet those demands. These resources include time, skills, knowledge and equipment.^17^

To improve the safe management of insulin during ToC, strategies must be targeted effectively to minimise misalignments between the demand and resources and enhance successful adaptations.^17^, ^18^ Therefore, it is essential that the work system is examined in detail to understand how different factors are contributing to outcomes.^19^, ^20^ The aim of this study is to map the processes of insulin management during transfers of care between primary and secondary care and the work system factors that impact them.

## 2 METHODS

### 2.1 Study design

We used an embedded case study approach,^21^ focusing on organisations within an integrated care system (ICS) in London. Qualitative methods were used to map insulin management during admission and following discharge for adults with type 2 diabetes mellitus. The focus on type 2 diabetes mellitus was due to the greater involvement of primary care teams in managing insulin for people with type 2 diabetes mellitus compared with type 1 diabetes. Approval was obtained from the NHS research ethics committee (Reference 22/EE/0155) as the study involved NHS patients and staff, and the university ethics committee (Reference 28148) for sponsorship. Informed consent was obtained for all participants. The use of multiple sources of data allowed a deeper understanding of how insulin is managed. It allowed comparison between guidelines (work as imagined), what was observed (work as done) and how those involved describe insulin management (work as disclosed).^21^

### 2.2 Data collection

Documentary analysis, observation and interviews were performed concurrently. Purposive, opportunistic observation was undertaken in a large teaching hospital based in London during 2022 and 2023. Eighty‐five hours were spent observing diabetes specialist nurses, nursing staff, clinical teams, pharmacists, discharge coordinators and PWDI undertaking insulin‐related activities within a large acute teaching hospital. The researcher (CL) used a contextual enquiry approach, aiming to be a detached observer, but questioning and seeking clarification to understand the activities being performed where required. Detailed field notes focusing on the people, tools, tasks and environments were taken and typed up as soon as possible.

Semi‐structured online interviews were held with 20 participants, see Table 1 for a breakdown of their roles in managing insulin. Participants represented different social and ethnicities; however, these data were not formally collected, and therefore quota sampling was not achieved. Healthcare professionals were initially recruited from a single ICS through connections made during observations or referral by healthcare professionals known to the researchers. Recruitment challenges were experienced within the ICS due to significant workforce pressures on staff and many PWDI declining to participate or being ineligible as they did not live within the ICS. Therefore, recruitment was expanded across England. Healthcare professionals were selected to represent a range of roles across care settings. PWDI were identified during their time in hospital by referral from the diabetes specialist nurses or pharmacists, or through invitations shared on national diabetes forums and on social media. PWDI or their caregivers were eligible if they were over 18, had type 2 diabetes mellitus, used insulin and had a hospital admission within the last 2 years. Interviews were held online for 30 minutes. Participants were asked to describe their experiences of managing insulin during admission and following discharge from hospital, to consider what helps safe insulin management and what challenges present during these times of transition. Interview transcripts were captured using video conferencing tools (Zoom and Microsoft Teams) and were updated for accuracy by the first author.

**TABLE 1:** Participants, role in insulin management and care setting.

Participant number	Role in managing insulin	Care setting at time of recruitment	Geographical location
1	Caregiver of person who uses insulin	Secondary care	London
2	Person who uses insulin	Primary care	South of England
3	General practitioner	Primary care	London
4	General practitioner	Primary care	London
5	General practitioner	Primary care	London
6	Diabetes specialist nurse	Primary care	London
7	Diabetes specialist nurse	Secondary care	South Central England
8	Medication safety pharmacist	Secondary care	South Central England
9	Emergency surgical unit pharmacist/Community pharmacist	Secondary care/primary care	South Central England
10	Surgeon	Secondary care	Midlands
11	Surgeon	Secondary care	Midlands
12	Diabetes specialist and emergency department nurse	Secondary care	London
13	Paramedic assistant	Primary care	South Central England
14	Primary care network Pharmacist/Community pharmacist	Primary care	South Central England
15	Primary care network pharmacist	Primary care	South Central England
16	Person who uses insulin	Secondary care	London
17	Person who uses insulin	Primary care	South Central England
18	Person who uses insulin	Primary care	Not disclosed
19	Person who uses insulin	Primary care	Midlands
20	Person who uses insulin	Primary care	Not disclosed

Local and national guidance relating to insulin management during ToC was identified using hospital intranet searches and through exploration of relevant organisational websites, including the Joint British Diabetes Societies for Inpatient Care Group (JBDS), the National Institute for Health and Care Excellence (NICE), the Royal College of General Practitioners (RCGP) and the Royal Pharmaceutical Society.

Full details of data sources are provided in the supplementary information.

### 2.3 Data Analysis

Thematic analysis was undertaken using a framework approach (pp. 219–262).^22^, ^23^ Field notes from observations, interview transcripts and documents were uploaded into NVivo 12 and analysed line by line.

The work system categories in the Systems Engineering Initiative for Patient Safety (SEIPS 101)^15^ tool were used as categories for the framework analysis. Factors were categorised as people, tasks, tools and equipment and environments (local, organisational and external). Further analysis of the data allowed the development of four sub‐themes relating insulin management during ToC and exploring how work system factors impacted these themes. Key components of the ToC process were also identified inductively.

The interviews, observations and data analysis were performed by CL, an experienced, female, medication safety pharmacist. The co‐authors included a second medication safety pharmacist who was also the digital clinical safety lead and a general practitioner (GP). The healthcare backgrounds of the authors allowed contextual insight into terminology and issues being observed and described. Themes and sub‐themes were developed iteratively through discussion with the co‐authors to enhance data validation.

## 3 RESULTS

**FIGURE 1:**
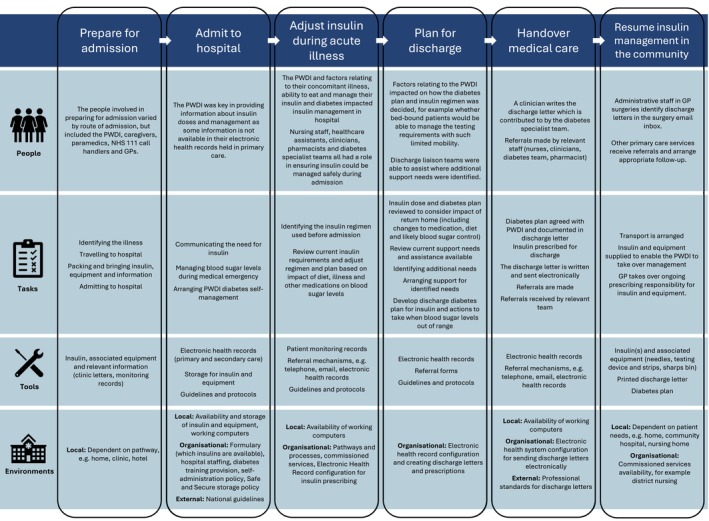
Work system factors identified for the different stages of insulin management during transfer of care.

Processes and system factors influencing insulin management during ToC between home and hospital were identified from policies and guidelines observation, and from interviews with PWDI, their caregivers and healthcare professionals. Six key stages of ToC were identified: preparing for admission, admitting to hospital, adjusting insulin during acute illness, planning for discharge, handing over medical care back to primary care and resuming insulin management in the community. Many system factors impacted these processes; see Figure 1 for a summary of SEIPS work system factors identified across the different ToC stages. Four key themes were identified as having the greatest potential to improve the safety of insulin as PWDI journeyed between hospital and home. The first was challenges around identifying, understanding, and adapting the PWDI's diabetes management plan. The second was recognising and incorporating the PWDI's expertise into insulin management. The third was the need for staff to be equipped with or have access to someone with the skills, knowledge and confidence to manage insulin. The final theme was the anticipation of potential issues after admission or following discharge and actions taken to prevent these.

### 3.1 Overarching factors that impacted all ToC stages

*The SEIPS components identified included external environment (winter pressures, industrial action), organisational environment (staffing, training) and tools (electronic health records, It hardware)*.

During the study period there were intense pressures on staff due to the impact of winter infections which led to high demand for hospital treatment alongside increases in staff absence due to illness. This was compounded by industrial action, both within the NHS and impacting national infrastructure (including railways and schools) further reducing staff availability. Healthcare professionals' capacity to manage insulin safely was consequently reduced, and activities were prioritised according to the urgency of clinical needs. For example, when the specialist diabetes team were short‐staffed, requests for advice were prioritised and other non‐urgent activities ceased to allow the team to manage their workload safely. Decisions about starting insulin or doses for discharge were made earlier, often based on more limited information, to allow education to be provided ahead of anticipated service disruption.

Electronic health records (EHR) were used extensively to manage insulin and communicate between healthcare professionals within and across different settings. The health information exchange (HIE) allowed read‐only access to medical notes from different care settings; for example, hospital staff could view general practice records and vice versa. Within general practice records, the PWDI's insulin information did not generally include dosing. Malfunctioning hardware or software often led to staff spending considerable time locating alternative computers to document plans, prescribe insulin and communicate with other staff members. Observation highlighted the diversity of PWDI who were admitted to hospital, and how multiple factors at individual, family and community levels were taken into consideration when optimising insulin management.

### 3.2 Information only available to PWDI

*The SEIPS components identified included people (presenting illness, co‐morbidities, ethnicity and religion), tasks (identifying need for admission, communicating) and tools (electronic health records, telephones, emails, insulin and diabetes records)*.

**FIGURE 2:**
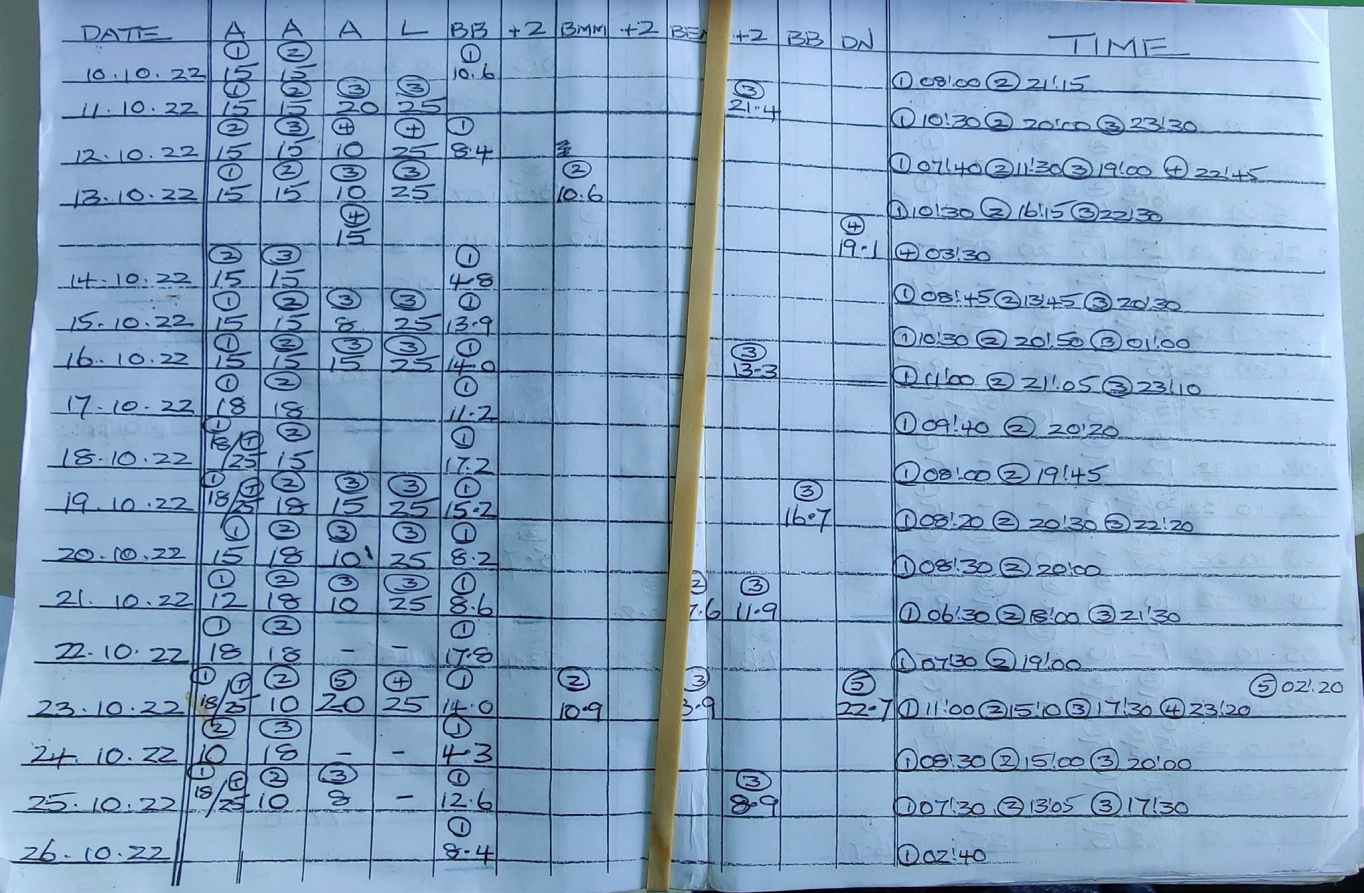
Patient started maintaining their own insulin monitoring and record book.

Once admitted to hospital, it was necessary to identify the insulin used by the PWDI and manage blood glucose levels in the context of the clinical situation. This required understanding the PWDI's usual insulin regimen and their diabetes management plan. These plans should include typical blood glucose ranges, and any adjustments or treatments for when levels are outside these ranges, or when the individual is unwell. The PWDI was often the only person with this information. Depending on the presenting illness, their ability to share this information varied. PWDI were not observed bringing in written diabetes plans, however interview participants described keeping information in a wallet or on medical alert bracelets. One PWDI was using a self‐designed record of blood glucose levels and insulin doses, however staff were unable to decipher the information it contained (see Figure 2). Challenges in identifying the PWDI's usual insulin regimens were described by interview participants.

The patient always feels to be the best source of information…for insulin, [digital] medical records are…very good at the brand, the device. [But] the major issue though for all of us is really the lack of information on dosing. Medication Safety Pharmacist (8)

National guidelines recommend the use of patient‐held insulin passports. These were not used widely in the hospital setting but were referred to by a community diabetes nurse.

So insulin passports… not really being used or don't know how well when they first come out, they were used. I don't know. Hospital‐based diabetes nurse (7)

Where PWDI brought information about their current diabetes plan, insulin regimen and monitoring records to hospital there was opportunity for the safe transfer of information to secondary care staff. PWDI expressed frustration that this key information was often not acted on or dismissed.

At the point I started having [a hyperglycaemic] episode…I was telling them “Something doesn't feel right and the last time that happened my blood glucose was all over the place”. And …they were like, “maybe it's just the anaesthesia wearing off”, and…they weren't listening to me. They assumed, they felt they knew better…and by the time I finally you know, got a nurse to check my blood sugar. It was skyrocketing, like it was critically high. PWDI (19)

PWDI also identified factors relating to their race and religion that impacted on how healthcare professionals behaved towards them and the likelihood of their requests or individual expertise in self management being acknowledged.

From my judgment it felt like as a person of colour, the attention I was given wasn't as much as I should have gotten. They just looked down, repulsively, I don't know. I try not to think about it much, it wasn't very pleasant of an experience. PWDI (20)

I know it's occupational hazard of what I wear [a religious headscarf], but sometimes it's like, I do not feel I get the empathy or the listening ear that I deserve, because I look or dress a certain way. PWDI (19)

### 3.3 Self management while in hospital

*The SEIPS components identified included people (PWDI skills, knowledge, nursing staff), tasks (risk assessments, administering, recording, monitoring), tools (insulin and monitoring equipment, electronic health records, insulin storage facilities) and environments (national policy, organisational policies)*.

Responsibility for managing diabetes and insulin during admission was generally held by nursing and medical staff. National guidelines recommend self management by PWDI in hospital as a key mechanism for improving safety. Enabling PWDI self management across ToC was viewed as challenging, requiring additional tasks for staff. Both assessing the ability of PWDI to self‐manage during inter‐current illness and putting in place practical arrangements to ensure insulin, monitoring equipment, and hypoglycaemia treatments were accessible were seen as key obstacles. These activities often did not happen. Staff and PWDI reported frustration with the situation.

I actually felt safer doing it myself than having a nurse do it, which is very odd. You should feel safer with the person that has more training, but ironically, your experience makes you feel safer than the person who has gone to…years of like nursing school. PWDI (20)

Hospital staff highlighted organisational policies requiring insulin be stored securely in a locked cupboard or refrigerator often prevented self‐administration.

Patients are getting frustrated that their insulin is being taken away from them, it's been locked away. Diabetes specialist nurse, hospital (7)

Furthermore, there were challenges uploading PWDI administration records onto the EHR. Therefore, staff were required to spend time transcribing this information onto the system on behalf of PWDI.

### 3.4 Confidence of staff in managing insulin

*The SEIPS components identified included people, tasks (reviewing insulin in the context of illness and adjusting dose, creating plans), tools (guidelines and electronic health records) and organisational factors (availability and provision of staffing and specialist roles, provision of training and supervision)*.

Optimising insulin dosage to reflect the current illness, concurrent medications and diet is critical for safety. With PWDI often excluded from this process either because of their condition or by organisational factors acting as barriers to self management, decision making mainly falls to healthcare staff. Frontline staff described ‘fear’ and ‘under‐confidence’ in managing insulin and often relied on the expertise of specialist diabetes teams to make decisions around suitable adjustments. Specialist teams struggled to cater for staff support needs due to their limited availability.

But there's a knowledge gap in that aspect…there was a bit of levity on their end, like, okay, they knew they have to [administer insulin], but they do not know why its so serious. PWDI (19)

We always, always, get the diabetic team involved…to review the patient and tell us exactly what we need to prescribe or not prescribe. Doctor (11)

National and organisational guidelines exist to support insulin adjustments decisions during acute illness, but specialist diabetes teams felt these were not always used in practice.

We've got these really good guidelines…but sometimes it's not followed. I don't know if it's because they ask the advice of the doctor, and the doctor just makes a number up or they think this is what should be or they don't read our notes [which explain the guidelines]. Hospital‐based diabetes specialist nurse (12)

### 3.5 Anticipation and prevention of potential ToC issues

*The SEIPS components identified included people (skills, experience and knowledge), tasks (planning admission needs, identifying discharge needs, making referrals), tools (patient held records, electronic health records, referral forms) and organisational factors (availability and provision of staffing and specialist roles)*.

Safe management of insulin across ToC required input from both the PWDI and a multidisciplinary team including community and hospital nurses, general practitioners, doctors, diabetes specialists and pharmacists among others. Many of those involved demonstrated their ability to recognise potential issues and act to avoid adverse outcomes. PWDI often informed their GP about changes to their diabetes management. One PWDI was so concerned about delays to his insulin when admitted to hospital that he arranged for his friend to bring his own supply from home.

Eventually I had to ask for insulin from home, and that took a while, but due to the delay and everything I had to do that. PWDI (20)

The specialist diabetes team used their skills and experience to proactively prevent problems occurring. They used their expertise to predict the impact of improving health, altered diet, changes in medication on PWDI blood glucose levels when deciding on insulin dosing for discharge.

Diabetes specialist nurses identified and addressed potential support needs PWDI might have after discharge. This was particularly important when needs changed during the admission. In one case, for a PWDI who had become bedbound during admission, the nurses considered whether insulin was still a suitable option given the challenges of administration and monitoring.

Once back in their own home, PWDI are likely to require adjustments in their insulin requirements; however, referral to the community diabetes team was not automatic. One primary care pharmacist described proactively following up PWDI following discharge:

…[PWDI's] needs change as soon as they…come out of hospital…generally I'll always…check if they're under the diabetes nurses locally… so they can have their insulin monitored. Primary Care pharmacist (14)

## 4 DISCUSSION

Many system factors impact insulin management during ToC. By mapping these in detail, potential areas for interventions to improve safety can be explored. Four themes that impacted the safe management of insulin during ToC were identified. The first of these was recognising and incorporating the expertise of PWDI in identifying diabetes management needs and ongoing insulin adjustments. Linked to this was enabling PWDI to manage their diabetes while in hospital. The third theme was the lack of confidence of healthcare staff in managing insulin. The fourth theme described the way in which the PWDI and their management team are involved in anticipating and proactively addressing potential challenges. We identified six processes as being key components of ToC for PWDI. These were preparing for admission, admitting to hospital, adjusting insulin during acute illness, planning for discharge, handing over medical care and resuming insulin management in the community. For each of these, multiple work system factors influenced how well these processes worked to ensure the PWDI were safely managed.

Much research around safe insulin management has been undertaken in hospital settings, and consequently interventions to improve safety have been mainly targeted in this sector.^24^, ^25^, ^26^ Maintaining safety for PWDI during ToC is an under‐researched area and this gap in evidence has been highlighted over many years.^10^, ^27^, ^28^ Using a complex work systems lens to identify the processes that impact insulin management in this context allows a detailed picture of the multiple interacting work system factors to be developed and, in doing so, presents new opportunities to improve and strengthen safety. Key work system factors that drive unsafe care include inadequate staffing, poor IT infrastructure, and the organisational and cultural factors that inhibit the expertise of the PWDI being recognised and applied. The mismatch between the demand placed on the system by ever‐increasing numbers of PWDI with complex needs and the availability of skilled, knowledgeable staff to manage the needs of PWDI is significant. This was demonstrated by the lack of confidence staff expressed in managing diabetes, and the over‐reliance on the limited capacity of the specialist diabetes teams. Maintaining safety is challenging when demand outstrips capacity.

Empowering PWDI throughout ToC is essential for safe diabetes management.^9^ PWDI potentially play a pivotal role in bridging safety gaps created by factors in the work system.^29^ Our study showed there is scope for improvement in incorporating their expertise during ToC. Supporting PWDI to provide sufficiently detailed information about their insulin and diabetes management in a format healthcare professionals can access could reduce the information gap during ToC. Patient held diabetic records have been proposed in the past. The Diabetes “Getting It Right First Time” report states that “electronic insulin passports, electronic patient records which include information on insulin needs, and electronic prescribing may also be effective in reducing insulin errors.”^24^ There is currently no standard template for such documents and previous attempts to introduce insulin passports have not been widely taken up.^30^ With the ongoing development of the NHS app,^31^ there may be potential for developing a shared diabetes records in the future.

Ensuring staff involved in managing insulin during ToC have access to the skills, knowledge and competency required is challenging. Current mechanisms to address this are aimed at the people within the work system through training and competency assessments and strengthening the support of specialist diabetes teams across organisations. Understanding how other aspects of the work system could be modified or re‐designed to support safe insulin management provides additional opportunities for improving safety.^32^ An example of a strengthened work system would be guidelines and procedures developed using human design principles to reduce the need to rely on memory.^33^ Mechanisms to incorporate key dosing information held by PWDI into EHR systems would strengthen the work system by providing access to essential information for healthcare staff.

### 4.1 Strengths and limitations

PWDI and a range of healthcare professionals were interviewed and observed to map work system factors influencing the safe management of insulin during ToC in real‐life situations while documentary analysis of relevant guidelines provided insight into the context of insulin management during ToC. The findings were validated through discussions within the study team, which brought together healthcare professionals from a range of backgrounds. Quota sampling for interview participants was not achieved; therefore, findings may not represent some aspects of healthcare inequalities.

Observation took place in one hospital within a single ICS, and resources, processes and guidelines will differ across the country. It was not possible to undertake observation within primary care; therefore, the factors that impact insulin management were gathered indirectly from interviews with both PWDI and healthcare professionals working in this setting. Observation of tasks performed in primary care would have strengthened understanding of the complexity of insulin management during ToC. Due to recruitment challenges, study participation had to be broadened to include NHS staff and PWDI from across England, making the case study unbounded. However, all participants described similar challenges and opportunities to those observed in the original ICS selected for the case study. This study focused on PWDI with type 2 diabetes. Our findings are likely to apply to any person with diabetes who uses subcutaneous insulin. For people with type 1 diabetes, there are even more factors likely to impact management due to additional technologies and more complex insulin regimens.

## 5 CONCLUSION

Managing insulin safely for PWDI during ToC is challenging due to the complexity introduced by all aspects of the work system. Current safety improvement mechanisms are often targeted at the people involved. Using a system‐based approach to discover the factors that impact safe management can identify additional opportunities to target safety improvement interventions for other aspects of the work system. Key areas for interventions are incorporating PWDI‐held information into EHR systems and developing mechanisms to support healthcare professionals apply insulin guidelines without relying on memory.

## FUNDING INFORMATION

This report is independent research funded by the National Institute for Health and Care Research ARC North Thames and was supported by the NIHR Oxford Biomedical Research Centre. The views expressed in this publication are those of the author(s) and not necessarily those of the National Institute for Health Research and Care or the Department of Health and Social Care.

## CONFLICT OF INTEREST STATEMENT

None to declare.

## Supporting information


Data S1:

## Data Availability

The data that support the findings of this study are available on request from the corresponding author. The data are not publicly available due to privacy or ethical restrictions.
