# Supplementary material for: Exploring the complexity of safe insulin management during transfer of care using qualitative methods
Source: Diabet Med. 2025 May 5;42(8):e70054. doi: 10.1111/dme.70054 (PMC12257437; doi:10.1111/dme.70054)
Supplement: Supplementary file 1 — Data S1: [file DME-42-e70054-s001.docx]

## Supplementary Information: Summary of data sources from fieldwork

Table 1: Documents identified for analysis

| Name of document | Published by | National/ Organisational or Local |
| --- | --- | --- |
| Simple steps to keep you safe during your hospital stay(130) | NHS England | National |
| Keeping patients safe when they transfer between care providers – getting the medicines right.(66) | Royal Pharmaceutical Society | National |
| Perioperative management of the adult surgical patient with diabetes. | Local organisation | Local |
| Diabetes at the front door(131) | Joint British Diabetes Societies for Inpatient Care Group | National |
| Discharge planning for adults with diabetes(132) | Joint British Diabetes Societies for Inpatient Care Group | National |
| Insulin essentials | Diabetes UK | National |
| Help with Hypos(133) | Novo Nordisk | National |
| Organisational procedures for prescribing insulin on the electronic health record | Local organisation | Organisational |

Table 2: Observation undertaken

| Date of observation | Area observed | Duration |
| --- | --- | --- |
| 18 Oct 2022 | Diabetes specialist teams | 6 hours |
| 21 Oct 2022 | Diabetes specialist teams | 6 hours |
| 2 Nov 2022 | Diabetes specialist teams | 6 hours |
| 11 Nov 2022 | Same day emergency care unit | 5 hours |
| 18 Nov 2022 | Diabetes specialist teams | 6 hours |
| 21 Nov 2022 | Diabetes specialist teams | 6 hours |
| 22 Nov 2022 | Pharmacist and Acute Medical Unit | 6 hours |
| 25 Nov 2022 | Pharmacist and Acute Medical Unit | 6 hours |
| 28 Nov 2022 | Diabetes specialist teams | 6 hours |
| 13 December 2022 | Acute Medical Unit | 4 hours |
| 9 February 2023 | Same day emergency care unit | 4 hours |
| 10 February 2023 | Same day emergency care unit | 4 hours |
| 2 March 2023 | Same day emergency care unit | 4 hours |
| 27 March 2023 | Acute Medical Unit and Same day emergency care unit | 3 hours |
| 29 March 2023 | Acute Medical Unit and Same day emergency care unit | 3 hours |
| 24 July 2023 | Diabetes specialist teams | 5 hours |
| 25 July 2023 | Diabetes specialist teams | 5 hours |

Table 3: Participants, role in insulin management and care setting

| Participant number | Role in managing insulin | Care setting at time of recruitment | Geographical Location |
| --- | --- | --- | --- |
| 1 | Caregiver of person who uses insulin | Secondary Care | London |
| 2 | Person who uses insulin | Primary Care | South of England |
| 3 | General Practitioner | Primary Care | London |
| 4 | General Practitioner | Primary Care | London |
| 5 | General Practitioner | Primary Care | London |
| 6 | Diabetes Specialist Nurse | Primary Care | London |
| 7 | Diabetes Specialist Nurse | Secondary Care | South Central England |
| 8 | Medication safety pharmacist | Secondary Care | South Central England |
| 9 | Emergency surgical unit pharmacist/Community pharmacist | Secondary Care/Primary Care | South Central England |
| 10 | Doctor | Secondary Care | Midlands |
| 11 | Doctor | Secondary Care | Midlands |
| 12 | Diabetes specialist and Emergency Department nurse | Secondary Care | London |
| 13 | Paramedic assistant | Primary Care | South Central England |
| 14 | Primary Care Network Pharmacist/Community pharmacist | Primary Care | South Central England |
| 15 | Primary Care Network Pharmacist | Primary Care | South Central England |
| 16 | Person who uses insulin | Secondary Care | London |
| 17 | Person who uses insulin | Primary Care | South Central England |
| 18 | Person who uses insulin | Primary Care | Not disclosed |
| 19 | Person who uses insulin | Primary Care | Midlands |
| 20 | Person who uses insulin | Primary Care | Not disclosed |
